# Takotsubo Cardiomyopathy: A Long Term Follow-up Shows Benefit with Risk Factor Reduction

**DOI:** 10.3390/jcdd2040273

**Published:** 2015-11-16

**Authors:** Koroush Khalighi, Mohammad Umar Farooq, Thein Tun Aung, Swe Oo

**Affiliations:** 1 Department of Cardiovascular Disease, Easton Hospital Easton, PA Clinical Professor of Medicine, Drexel University, School of Medicine Easton Cardiovascular Associates 2001 Fairview Ave, Easton, PA 18042, USA; 2 Department of Medicine, Easton Hospital, 250 South 21 street, Easton, PA 18042, USA; E-Mail: Mohammad_Farooq@chs.net; 3 Department of Cardiovascular Disease, Wright State University, 921 Wilmington Ave, Apt D, Dayton, OH 45420, USA; E-Mail: theintun.aung@hotmail.com; 4 Department of Medicine, Easton Hospital, 250 South 21 street, Easton, PA 18042, USA; E-Mail: swezin82@gmail.com

**Keywords:** Takotusbo Cardiomyopathy (TC), Myocardial Infarction with Normal Coronary Arteries (MINCA), stress-induced cardiomyopathy, Coronary Artery Disease (CAD)

## Abstract

Only sparse data was available on long-term of Takotusbo Cardiomyopathy (TC). Previous studies suggested prognosis is not necessarily benign. We report the long-term follow-up of 12 TC patients actively managed with risk factor reduction. Retrospective analysis of all patients diagnosed with TC at our hospital between 1998 and 2010. We identified 12 patients with TC among 1651 cases of emergent left heart catheterization over 12 years. Mean follow-up time was 8.3 ± 3.6 years. All were female, 87% had hypertension, 25% had history of Coronary Artery Disease (CAD), 67% had hyperlipidemia, 44% had some preceding emotional trauma, and 44% had some physical/physiological stress. Previous studies have shown that over 50% of TC patients experience future cardiac events, and 10% have a recurrence of TC. Patients were prescribed therapeutic lifestyle changes (TLC) and guideline directed medical therapy (GDMT) for aggressive risk factor reduction. TLC included diet, exercise, and cardiac rehabilitation. GDMT often included aspirin, beta-blockers, ACE-inhibitors, and statins. Follow-up echocardiograms showed recovery and maintenance of the ejection fraction. There was no cardiac mortality and no recurrences of TC. Aggressive risk factor reduction with TLC and GDMT may be effective in improving the long term outcomes of patients with TC.

## 1. Introduction

Takotsubo Cardiomyopathy (TC) was first described in 1991 in Japan. It consists of transient systolic dysfunction of the apical and/or mid-segment of the left ventricle, a clinical presentation of that mimics acute myocardial infarction and absence of obstructive coronary artery disease. Sparse data available on long-term outcomes suggests prognosis is not necessarily benign. We report the long-term follow-up of 12 TC patients, and speculate that intensive risk factor modification may account for the good outcomes we observed in this group.

## 2. Methods

From January 1998 to December 2010, among 1651 who underwent emergent left heart catheterization for acute coronary syndrome (ACS), 12 patients were identified with having TC using the Modified Mayo Criteria. Main components include akinesia of mid-distal LV, non-obstructive Coronary Artery Disease (CAD), new EKG changes, and absence of myocarditis or pheochromocytoma (Table 1).

**Table 1:** Demographic Characteristics.

Patient Number	Follow up (Years)	Age (Year)	Sex	HTN	DM	CAD	Dyslipidemia	FH CAD
A	13	57	F	No	No	No	Yes	Yes
B	17	36	F	No	No	No	Yes	Yes
C	9	67	F	No	No	No	No	No
D	8	72	F	Yes	No	No	Yes	No
E	7	61	F	No	No	Yes	Yes	No
F	10	89	F	Yes	No	No	No	No
G	6	41	F	Yes	No	No	No	No
H	6	69	F	Yes	No	Yes	Yes	Yes
I	5	69	F	Yes	No	Yes	Yes	Yes
J	5	81	F	Yes	Yes	No	Yes	No
K	5	56	F	Yes	Yes	No	Yes	Yes
L	8	65	F	Yes	No	Yes	Yes	Yes
			100% F	75%	17%	25%	67%	50%

All patients underwent follow-up evaluation of left ventricular function with transthoracic echocardiogram six weeks after the initial event, followed by yearly echocardiograms. We followed these 12 patients, who were actively managed with both lifestyle modification and pharmacologic treatment, over the 5–17 years period after the initial TC event.

Patients were advised regarding therapeutic lifestyle changes (TLC) and prescribed guideline-directed medical therapy (GDMT) for aggressive risk factor reduction. TLC included low-salt, low-fat diet, exercise, and cardiac rehabilitation. GDMT often included anti-platelet agents (aspirin, clopidogrel), beta-blockers (metoprolol or carvedilol), angiotensin converting enzyme inhibitors (ACEI) (lisinopril or ramipril), and statins (atorvastatin). The primary end point was a combination of all-cause death, cardiogenic shock, sudden cardiac death, recurrence of TC, and re-hospitalization for cardiac reasons.

## 3. Results

All of the patients in our study were female and most were post-menopausal with a median age of 66 years (ranging from 36 to 89 years). Many had risk factors for coronary artery disease (CAD) with 75% (9/12) having hypertension, 67% (9/12) with hyperlipidemia, and 50% (6/12) with a family history of CAD. There were 25% (4/12) who already had a documented history of CAD. On presentation, 58% (7/12) had preceding emotional trauma and 50% (6/12) had physical stress (Table 1 and Table 2). All patients presented with chest pain and troponin elevation, while 50% (6/12) had ST elevations on EKG. Coronary angiography revealed no obstructive lesion and left ventriculography revealed apical hypokinesis along with decreased ejection fraction (EF) in all patients (Table 2).

**Table 2:** Clinical Features.

Patient	Psychological Trauma	Physical Stress	Chest Pain	EKG	Peak Troponin Elevation	Hypokinesis on Ventriculography
A	Yes *	Yes ^#^	Substernal	STE	2.4	Apical
B	Yes ^	No	Substernal	STD-inferior	42	Inferior septal wall
C	Yes	No	Substernal	STE	1.8	Apical
D	Yes	No	Substernal	None	1.4	Infero-apical
E	Yes	Yes	Substernal	None	10	Apical
F	No	Yes	Left Sided	None	1.85	Apical
G	No	Yes	Substernal	STE-inferior	4.13; 1.79	Infero-apical
H	No	Yes	Substernal	STE-anterior	1.39	Infero-apical
I	No	Yes	Left sided	LBBB	1.37	Apical
J	Yes	No	Substernal	STE-anterior	1.45	Apical
K	Yes	No	Substernal	T-inv-inferior	3.34	Infero-apical
L	Yes	No	Substernal	T-inv-inferior	1.5	Infero-apical
				ST elevation = 50%		

* = Argument at work; ^ = Congenital problem in son; ^#^ = yard work.

The patients in our study have been followed for 5 to 17 years, with an average follow-up of 8.3 ± 3.6 years. In all patients, echocardiograms performed four weeks after the initial event demonstrated recovery of EF along with normalization of cardiac contraction (Table 3). Further yearly follow-up echocardiograms showed that patients also maintained their ejection fraction for the duration of the study.

**Table 3:** Management and follow up.

Patient	Initial Ejection Fraction (Percentage)	Follow-up Ejection Fraction (Percentage)	Administered Medications	Follow up (Years)	Chest Pain Recurrence	Documented CAD on Follow-up
A	45	70	Aspirin, Metoprolol, Pravastatin Ramipril	13	Yes	No
B	50	60	Metoprolol, Aspirin	17	No	No
C	25	40	Aspirin, Metoprolol, Clopidogrel, Ramipril	9	No	No
D	35	55	Aspirin, Lisinopril, Metoprolol, Clopidogrel	8	No	No
E	35	55	Metoprolol, Atorvastatin	7	No	No
F	35	61	Lisinopril, Carvedilol	10	Yes	No
G	50	55	Aspirin, Clopidogrel, Valsartan, Atorvastatin	6	Yes	No
H	35	60	Aspirin, Clopidogrel, Metoprolol, Statin	6	No	No
I	25	45	Aspirin, Lisinopril, Metoprolol, Statin,	5	No	No
J	40	55	Aspirin, Statin, Lisinopril, Amlodipine	5	No	No
K	30	50	Aspirin, Metoprolol, Statin, Lisinopril	5	No	No
L	25	60	Aspirin, Statin, Carvedilol, Fosinopril **	8	Yes	Yes

** = Progression of CAD from 30% stenosis of RCA that was later found to be 90%.

There were four patients who experienced recurrences of chest pain. Two patients’ symptoms were musculoskeletal in etiology. One patient had a progression of CAD, which was managed conservatively. One patient was found to have a malignant left lung mass. There was no cardiac mortality, and no recurrences of TC.

We followed the blood pressure, heart rate, body weight, and lipid panel results to ensure the patients are following optimal medical therapy and lifestyle modifications. The results are presented in Table 4 and Table 5. During the follow up, one patient passed away with lung cancer. Her results will not be updated in the following tables.

After optimal, guideline-directed medical therapy (GDMT), almost all the patients had their blood pressure and heart rate controlled. The majority (8/11) of the patients had maintained or reduced body weight. The majority of the patients (9/11) had well-controlled LDL levels. The LDL level of patient A and patient D had worsened recently. Further review revealed that before these changes, their medical insurances were switched under the new healthcare law. Rosuvastatin was switched to generic Pravastatin and Atorvastatin. Follow up lipid panel results are pending.

**Table 4:** Changes in blood pressure, heart rate, and body weight with medical management.

Patient	Systolic Blood Pressure at Diagnosis	Most Recent Systolic Blood Pressure	Heart Rate at Diagnosis	Most Recent Heart Rate	Body Weight at Presentation	Most Recent Body Weight
A	122	132	62	60	144	186
B	132	120	83	60	142	135
C	124	120	72	70	156	147
D	193	120	74	66	99	97
E	130	128	68	65	172	190
F	156	104	74	75	130	135
G	140	111	72	84	115	111
H	116	106	117	98	182	145
I	167	141	85	83	266	266
J	131	128	62	72	149	140
K	135	128	102	80	356	270
L	117	125	90	78	164	175

**Table 5:** Lipid panel results after medical management.

Patient	Initial Cholesterol	Most Recent Cholesterol	Initial LDL	Most Recent LDL	Initial HDL	Most Recent HDL	Initial Triglycerides	Most Recent Triglycerides
A	169	197	71	112	59	53	124	159
B	164	140	80	60	65	66	91	71
C	159	145	90	73	61	58	76	86
D	164	202	87	127	60	59	85	77
E	212	154	135	95	52	56	168	204
F	153	155	101	93	37	46	72	80
G	168	171	73	79	82	79	61	64
H	172	142	113	63	19	26	202	173
I	168	154	99	81	32	36	187	183
J	151	121	73	59	30	19	238	202
K	154	180	66	74	36	58	258	302
L	119	107	54	37	50	35	75	174

LDL = Low Density Lipoprotein; HDL = High Density Lipoprotein.

## 4. Discussion

TC is increasingly recognized in the differential diagnosis of acute coronary syndrome (ACS) as a cause of myocardial infarction with normal coronary arteries (MINCA) [1]. Numerous studies document the short-term recovery of TC patients. A meta-analysis of 28 case series by Pilgrim *et al.*, showed complete recovery of EF in 95.9% of patients in 7–37 days, with ejection fraction improving from 20%–49.9% to 59%–76% [2]. Few studies, however, followed the long-term course of these patients.

The few studies following long-term outcomes in TC patients arrive at differing conclusions regarding prognosis. Elesber *et al.*, and Valbusa *et al.*, both tracked overall survival in age and gender-matched TC populations, but found no difference. In contrast, Sharkey *et al.*, and Ionesco *et al.*, found that survival in TC patients was reduced [3,4,5,6]. Importantly, all of the aforementioned studies showed high cardiovascular event rates following the initial insult. In the follow-up by Elesber *et al.* during the 4.7 ± 4.8 years follow-up, 17% of patients suffered mortality, 10% of patients suffered recurrence of TC, and 12% of patients suffered re-hospitalization for cardiac reasons, for a total 39% event rate [5]. Ionesco *et al.* also saw this high event rate in their follow-up of 27 TC patients over a mean of 27 ± 16 months. 52% of patients reached a primary end point of a combination of all-cause death, cardiogenic shock, sudden cardiac death, and re-hospitalization for cardiac reasons, with the authors concluding that the long-term outcome of TC is worse than previously reported [3]. In a recent study, spectroscopy and imaging done four months after the initial TC event showed cardiac energetic impairment, adding more evidence of the long term impairment in health and well-being of TC patients [7].

In a recent prospective study by Looi *et al.*, 26% of patients had a complicated hospital course, 4% need circulatory support with an intra-aortic balloon pump, 6% need ventilatory support, 2% in-hospital mortality, and an additional 4% mortality soon after hospital discharge [8].

Meta-analysis by Singh *et al.*, 2014, showed that the in-hospital mortality rate of TC was 4.5%, but only 38% of the mortality was directly related to TC and complications. The remaining 62% of the mortality was due to underlying non-cardiac mortality. It brought the cardiac mortality from TC down to 1.5% [9].

In comparison, we found a trend towards a decrease in morbidity and mortality of TC patients who were prescribed cardiac medical therapy on discharge, indicating a possible benefit from a long-term management strategy (Table 6).

**Table 6:** Comparing the Data from Previous Studies.

	Ionesco *et al.*	Elesber *et al.*	Khalighi *et al.*	Looi *et al.* 2012
Number of Patients	27	100	12	100
Mean Age (years)	68 ± 14	66 ± 13	64 ± 15	65 ± 11
Follow/up Duration	2.25 ±1.3 years	4.7 ±4.2 years	8.3 ± 3.6 years	3.0 ±1.7
Patients Antiplatelet agent	33%	50%	83%	90
Patients on BB	33%	51%	83%	65%
Patients on ACE I/ARB	41%	74%	83%	63%
Patients on Statin	30%	32%	67%	69%
Overall patients on optimal medical therapy	34.3%	51.8%	79.0%	
Results				
Chest pain	2	31	4	78%
Hospitalization for cardiac complaints	8	12	1	26%
Recurrence of TC	2	10	0	7 (7%)
Mortality	4	17	0	4 (4%)
Morbidity/Mortality	14 (52%)	29 (29%) *	1 (8%)	26 (26%)

Morbidity and mortality = hospitalization for cardiac reasons, death from any cause; * event rate is 39% if we include recurrence of TC.

Medical therapy was targeted at preventing the mechanisms believed to cause TC:

(1) Cardioselective beta blockers (carvedilol, metoprolol) to prevent myocardial stunning or microinfarction caused by catecholamine-induced sympathetic overdrive: Wittstein *et al.*, showed that catecholamine levels are 2–4 times higher in patients with stress-induced cardiomyopathy as compared to patients with myocardial infarction. [10] This is the most widely accepted mechanism leading to TC [10,11].
(2) Cardioselective beta-blockers, Calcium channel blockers and/or ACE-I/ARB may prevent coronary artery spasm—Lacy *et al.* [12] showed that stress from public speaking can lead to vasoconstriction of coronary artery segments. Kiuriso *et al*. demonstrated that three of 30 patients with TC had spontaneous multivessel epicardial coronary spasm on angiography, and 10 of 14 patients with TC had coronary spasms on a provocation test using either egonovine or acetylcholine.
(3) Statins and anti-platelet agents to prevent microvascular dysfunction leading to transient thrombosis [2,13]. In a study of 16 patients with TC, Bybee showed that all 16 patients had decreased coronary blood flow in at least one epicardial vessel and 10 patients had this in all three epicardial vessels [14]. In one of the earlier studies of TC, Sadamatsu *et al.* [13] studied the angiography of two patients with TC and found that both had reduced coronary flow reserve.

In cross-analysis with TC patients, followed long-term in other studies, we observed a negative correlation between the percentage of patients on cardiac medications and the subsequent adverse event rate. In contrast to previous studies, most patients in our study were placed on appropriate cardiac medications at the time of discharge (Table 6). They maintained their ejection fraction with only one patient requiring readmission for progression of CAD. Over a long period of follow-up, our patients did well with medical management, including TLC and GDMT. This suggests that aggressive risk factor reduction by medical management might explain the difference in long term prognosis of TC patients seen in these studies.

## 5. Limitations

This is a single center trial with a limited number of patients without any control group. However, our study has the longest documented follow-up to prove the benefit of long-term medical management. However, due to limited power, we cannot specify which medication produced the greatest benefit.

Our results might also be compounded by selection bias since we followed 12 patients with Takotsubo Cardiomyopathy after discharge from hospital. We selected only the survivors. All of our patients survived the initial hospitalization. The biggest limiting factor to any conclusive statement regarding long-term prognosis and management is the relatively small number of patients available for such an analysis. As the diagnosis becomes more prevalent, future studies may be able to access a larger patient population for greater power.

## 6. Conclusions

TC patients may be at risk of experiencing long-term cardiovascular events. Following 12 TC patients with TLC and GDMT, we observed recovery and maintenance of the ejection fraction, no cardiac mortality, and an excellent long-term prognosis. Risk factor reduction with TLC and GDMT may be effective in reducing the risk of long-term cardiovascular morbidity and mortality in TC patients.

## References

[1] Seth P.S., Aurigemma G.P., Krasnow J.M., Tighe D.A., Untereker W.J., Meyer T.E. (2003). A syndrome of transient left ventricular apical wall motion abnormality in the absence of coronary disease: A perspective from the United States. Cardiology.

[2] Pilgrim T.M., Wyss T.R. (2008). Takotsubo cardiomyopathy or transient left ventricular apical; ballooning syndrome: A systematic review. Int. J. Cardiol..

[3] Ionescu C.N., Aguilar-Lopez C.A., Sakr A.E., Ghantous A.E., Donohue T.J. (2010). Long-term outcome of Tako-tsubo cardiomyopathy. Heart Lung Circ..

[4] Valbusa A., Abbadessa F., Giachero C., Vischi M., Zingarelli A., Olivieri R., Visconti L.O. (2008). Long-term follow-up of Tako-Tsubo-like syndrome: A retrospective study of 22 cases. J. Cardiovasc. Med..

[5] Elesber A.A., Prasad A., Lennon R.J., Wright R.S., Lerman A., Rihal C.S. (2007). Four-year recurrence rate and prognosis of the apical ballooning syndrome. J. Am. Coll. Cardiol..

[6] Sharkey S.W., Windenburg D.C., Lesser J.R., Maron M.S., Hauser R.G., Lesser J.N., Haas T.S., Hodges J.S., Maron B.J. (2010). Natural history and expansive clinical profile of stress (tako-tsubo) cardiomyopathy. J. Am. Coll. Cardiol..

[7] Dawson D.K., Neil C.J., Henning A., Cameron D., Jagpal B., Bruce M., Horowitz J., Frenneaux M.P. (2015). Tako-Tsubo Cardiomyopathy: A Heart Stressed Out of Energy?. JACC Cardiovasc. Imaging.

[8] Looi J.L., Wong C.W., Khan A., Webster M., Kerr A.J. (2012). Clinical characteristics and outcome of apical ballooning syndrome in Auckland, New Zealand. Heart Lung Circ..

[9] Singh K., Carson K., Usmani Z., Sawhney G., Shah R., Horowitz J. (2014). Systematic review and meta-analysis of incidence and correlates of recurrence of takotsubo cardiomyopathy. Int. J. Cardiol..

[10] Wittstein I.S., Thiemann D.R., Lima J.A., Baughman K.L., Schulman S.P., Gerstenblith G., Thiemann D.R., Lima J.A., Baughman K.L., Schulman S.P. (2005). Neurohumoral features of myocardial stunning due to sudden emotional stress. N. Engl. J. Med..

[11] Coupez E., Eschalier R., Pereira B., Pierrard R., Souteyrand G., Clerfond G., Citron B., Lusson J.R., Mansencal N., Motreff P. (2014). A single pathophysiological pathway in Takotsubo cardiomyopathy: Catecholaminergic stress. Arch. Cardiovasc. Dis..

[12] Lacy C.R., Contrada R.J., Robbins M.L., Tannenbaum A.K., Moreyra A.E., Chelton S., Kostis J.B. (1995). Coronary vasoconstriction induced by mental stress (simulated public speaking). Am. J. Cardiol..

[13] Dorfman T.A., Iskandrian A.E. (2009). Takotsubo cardiomyopathy: State-of-the-art review. J. Nucl. Cardiol..

[14] Bybee K.A., Prasad A., Barsness G.W., Lerman A., Jaffe A.S., Murphy J.G., Wright R.S., Rihal C.S. (2004). Clinical characteristics and thrombolysis in myocardial infarction frame counts in women with transient left ventricular apical ballooning syndrome. Am. J. Cardiol..

